# Supplementary figures and images for: Protocol for evaluating the effects of integrating music with taekwondo training in children with autism spectrum disorder: A randomized controlled trial
Source: PLoS One. 2025 Jan 31;20(1):e0315503. doi: 10.1371/journal.pone.0315503 (PMC11785272; doi:10.1371/journal.pone.0315503)

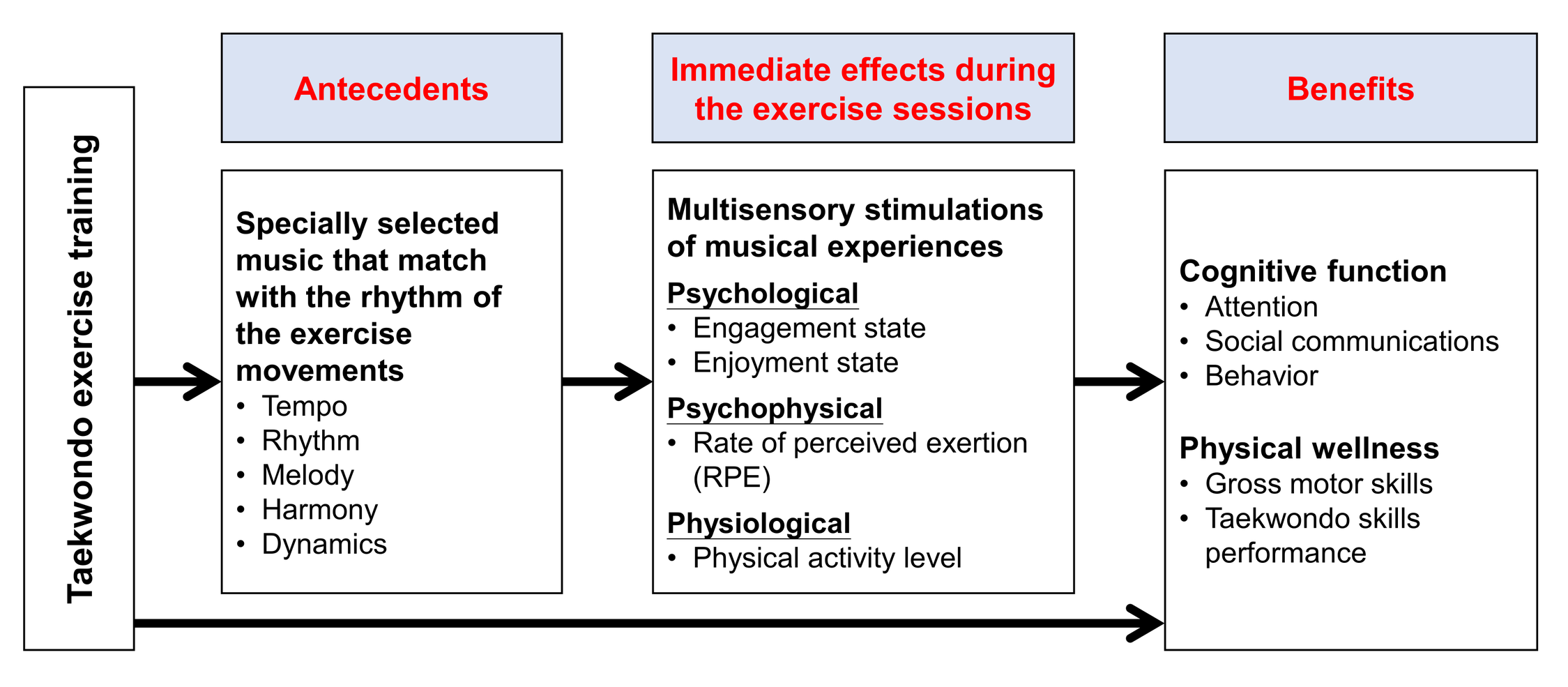

Supplement: S1 Fig — (TIF) [file pone.0315503.s002.tif]
